# A Giant Cell Tumor of the Tendon Sheath of the Foot: A Rare Presentation

**DOI:** 10.7759/cureus.63276

**Published:** 2024-06-27

**Authors:** Akash Inamdar, Rajesh G Gattani, Raju K Shinde

**Affiliations:** 1 General Surgery, Jawaharlal Nehru Medical College, Datta Meghe Institute of Higher Education and Research, Wardha, IND

**Keywords:** tumor surgery, excision of the tumor, giant cell tumor of the tendon sheath, giant cell tumor, foot tumor

## Abstract

A giant cell tumor of the tendon sheath (GCTTS) is a benign tumor that can occur in the joint synovium, bursae, or tendon sheath. It generally emerges in the tendons/synovium of the bones of the hand. It has unique characteristics, as noted in histopathological, clinical, and published literature. GCTTS has been reported across different age groups, with higher incidence observed in middle-aged adults. We present an unusual occurrence of GCTTS arising from the foot in a 54-year-old female who visited our medical facility with a history of swelling in her right foot for one year. Ultrasonography suggested a well-defined 5 x 4 cm lesion deep to the flexor tendon with possible intertarsal extension, which was managed surgically. This article represents a detailed understanding of GCTTS, emphasizing its benign yet locally aggressive nature and the complexities involved in its diagnosis and management.

## Introduction

The giant cell tumor of the tendon sheath (GCTTS) is a non-malignant proliferative lesion that can occur in the soft tissues, such as the tendon sheath, bursae, or joint synovium. It was first termed by Jaffe and his collegues in 1949, following the initial description by Chassaignac in 1852. GCTTS is known for its local aggressiveness. It can be challenging to treat due to the delicate balance required between removing the tumor entirely and preserving the adjoining vital tissue structures such as nerves and tendons [1]. GCTTS is considered the second most common benign type of tumor, generally occurring in hands after ganglion cysts [2]. These tumors usually are slow-growing and often found on the tendon sheath. They are more frequently observed in adults between the ages of 30 and 50 years, and there seems to be a higher incidence in females [3]. Despite being benign, they can recur after surgical removal, with recurrence rates reported as high as 15-45% in some studies. The diffuse type is prone to recur as compared to the localized type [4], though another study found a lower recurrence rate of 6%. A high recurrence rate is noted in the tumors located in the vicinity of the interphalangeal joints. Magnetic resonance imaging has been recommended for diagnostic and therapeutic purposes. It can provide detailed images of the tumor's size, shape, and its relationship to surrounding structures, including whether there has been penetration into joints or bone cortices or involvement of neurovascular bundles [5]. Treatment usually involves surgical excision, and careful removal is necessary to preserve functionality and reduce the recurrence rate. After surgery, patients typically have a good prognosis and can often retain a range of motion comparable to the contra-lateral hand [6].

## Case presentation

**Figure 1:**
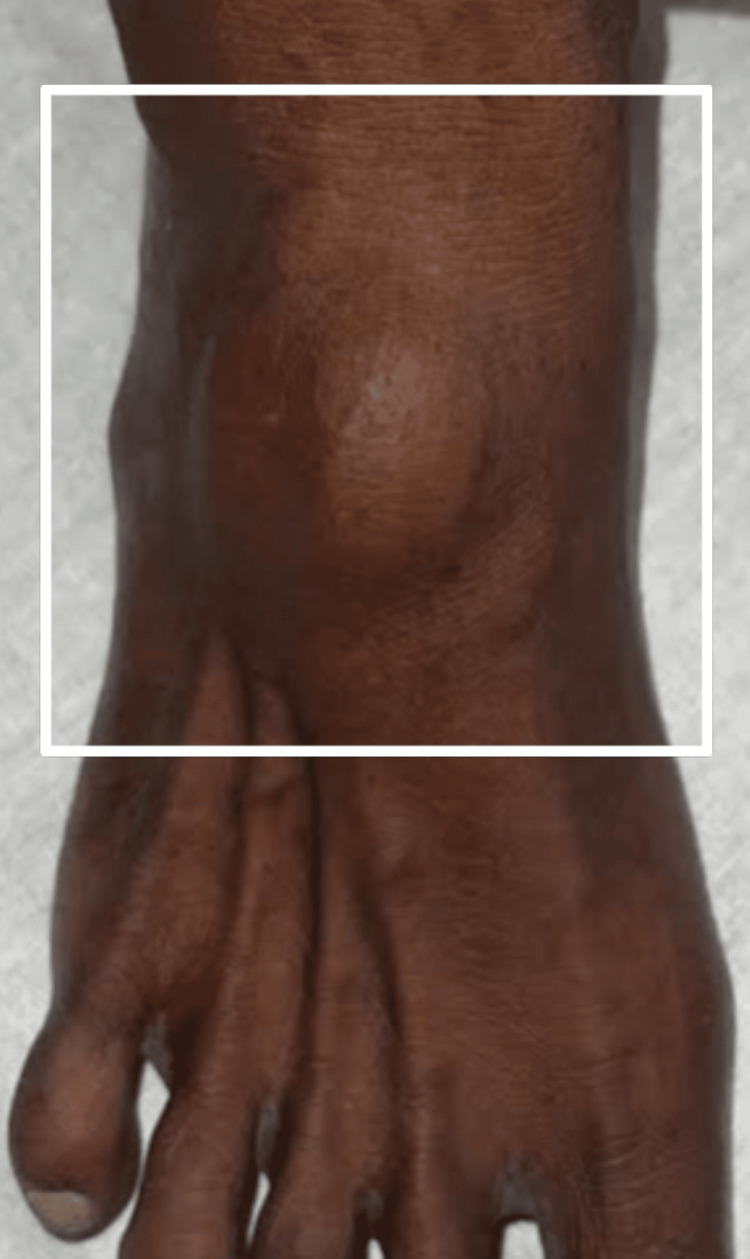
Preoperative clinical photograph in a front view

**Figure 2:**
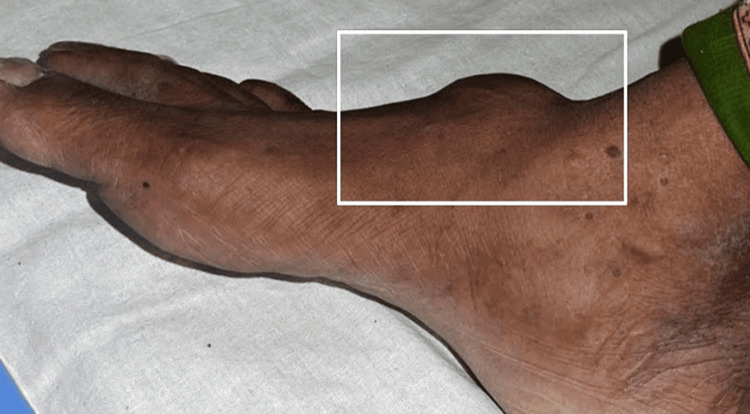
Preoperative clinical image of the side view

A 54-year-old female came to the Outpatient Department with a chief complaint of swelling over her right foot for one year. She had a solitary swelling, which was insidious in onset and gradually progressive in nature, associated with pain while walking and a tingling sensation in the foot. No history of fever, loss of power in the feet, weight loss, or appetite was noted. She had no similar history in the past or similar swellings anywhere in her body. She did not have any comorbidities and denied any addictions. On examination, it was a 5 x 4 cm single, well-defined swelling with smooth margins, soft to firm, and uniform in consistency, with little side-to-side mobility, no rise in temperature, and normal skin over the swelling. It was located about 2.5 cm distal to the right ankle joint on the dorsal surface. But on dorsiflexion against the resistance, the swelling became more prominent and fixed to the underlying structures, as shown in Figures 1-2.

**Figure 3:**
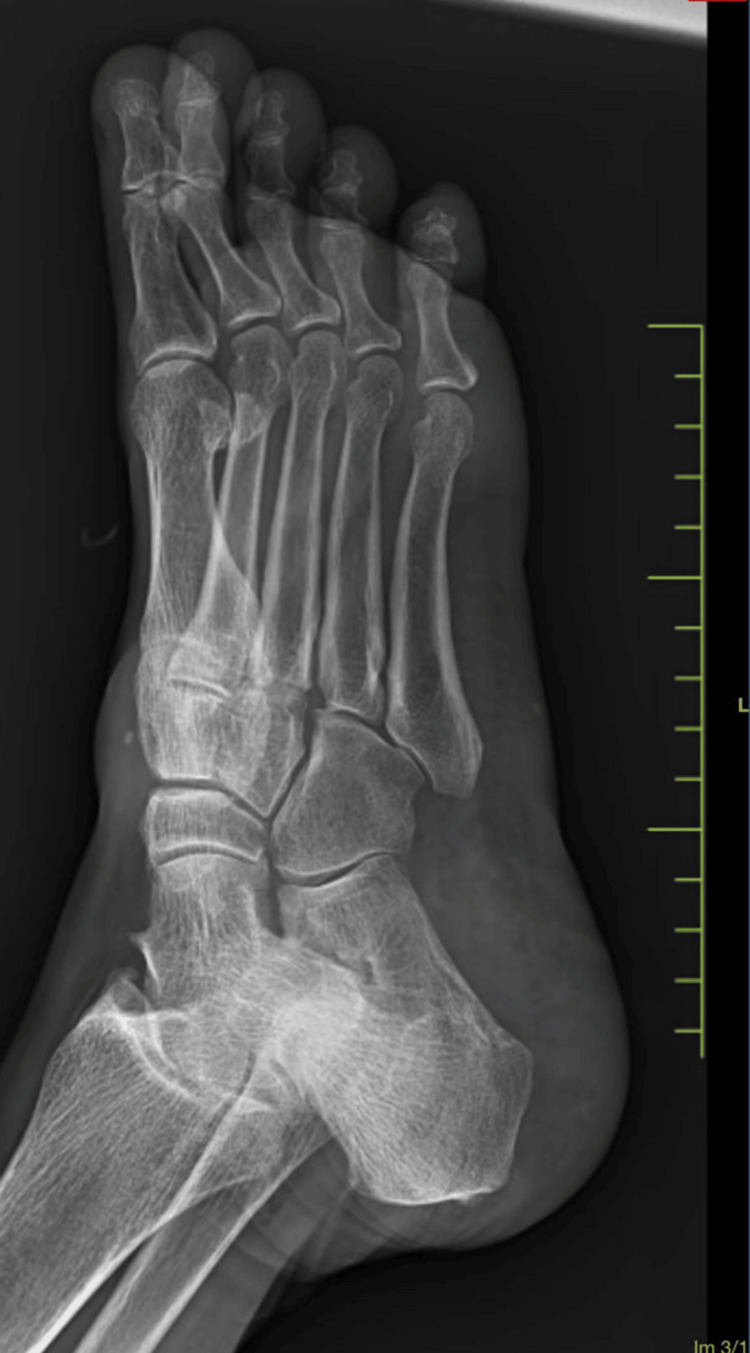
X-ray image of the foot (oblique view)

**Figure 4:**
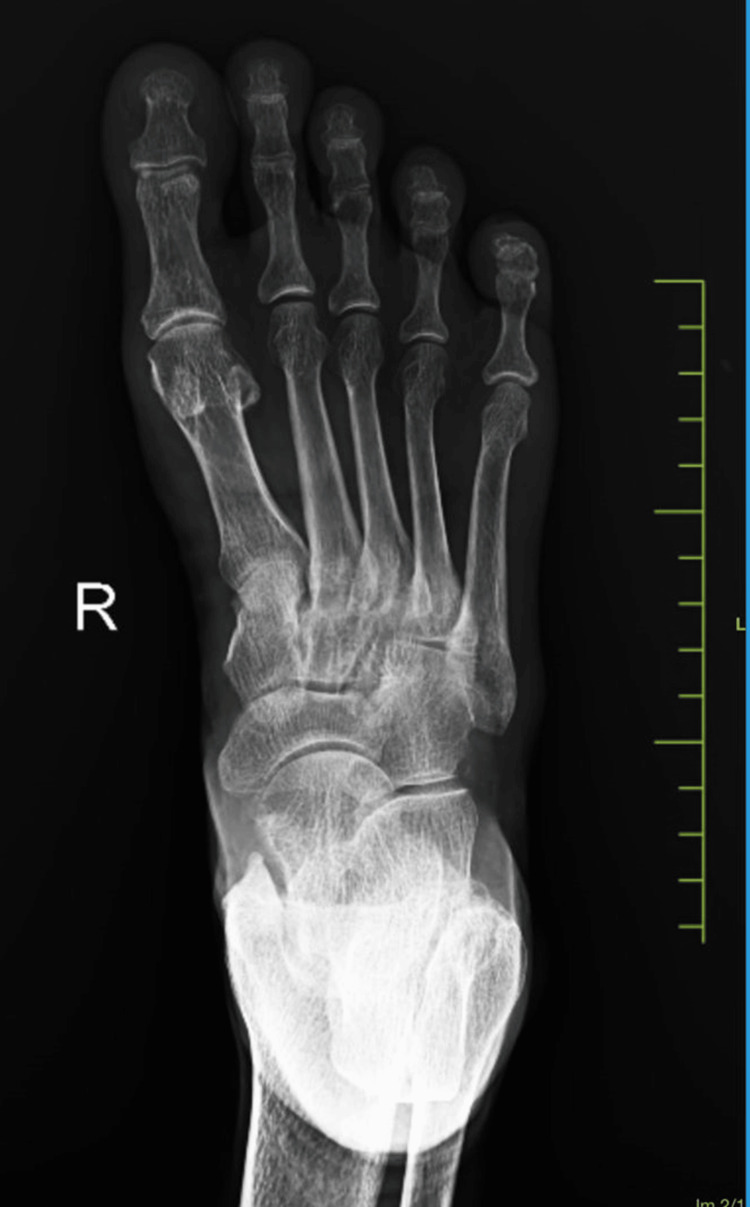
X-ray image of the foot (anteroposterior view)

The blood investigations were within the normal limits. Ultrasound imaging of the swelling was suggestive of a well-defined, heterogeneously hypoechoic lesion in the dorsal aspect of the right foot at the tarsal region, superficial to the underlying bone and deep to the flexor tendons, measuring approximately 52.2 x 42 x 12.8 mm, showing minimal vascularity with possible intertarsal extensions likely to be neoplastic. X-ray of the right foot showed no evidence of bone involvement and/or erosion (Figures 3-4).

**Figure 5:**
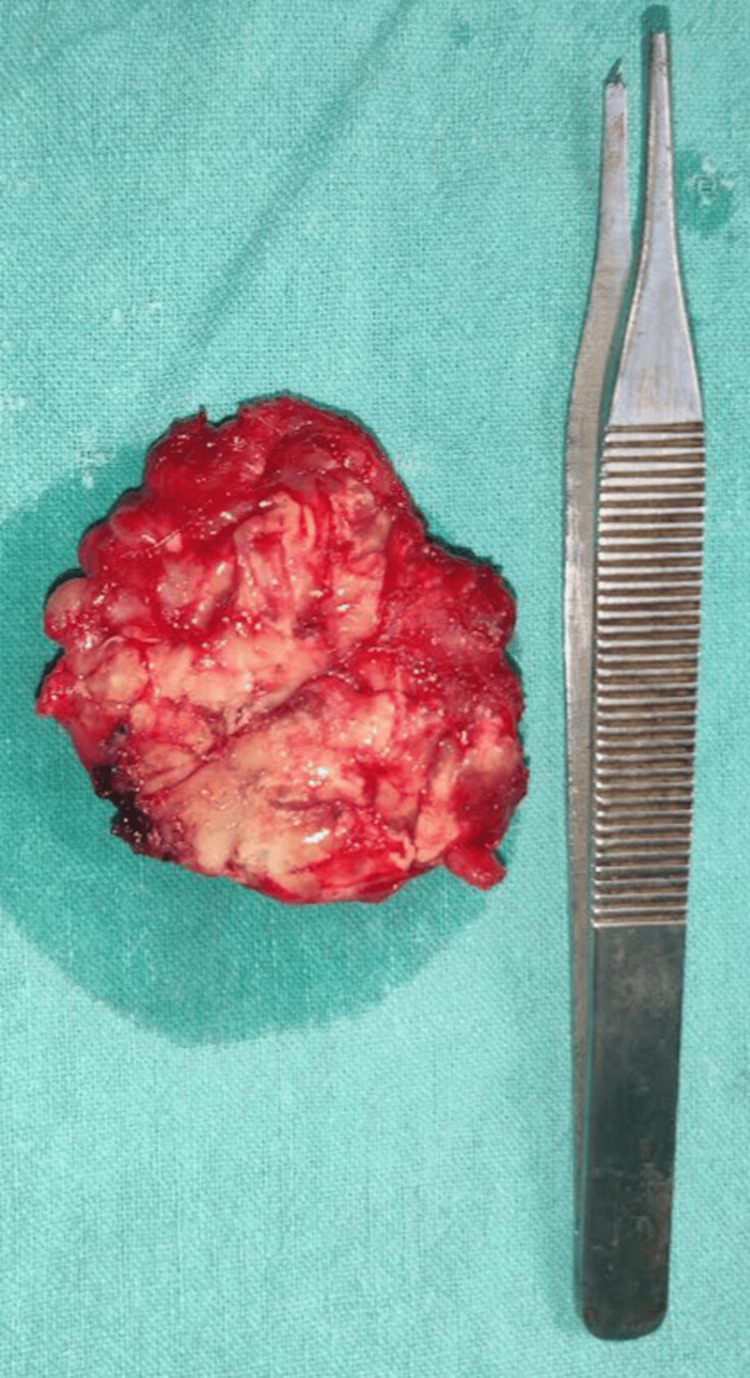
In toto excised specimen

The patient was planned for elective surgical excision of the swelling over the foot at the ankle block. Intraoperatively, swelling arose within the flexor digitorum brevis with extensions into adjacent muscles and tendons. The capsule of the swelling was adherent to the underlying tarsal bones. No obvious osseous connection was present. The specimen was excised in toto with its capsule, as shown in Figure 5.

**Figure 6:**
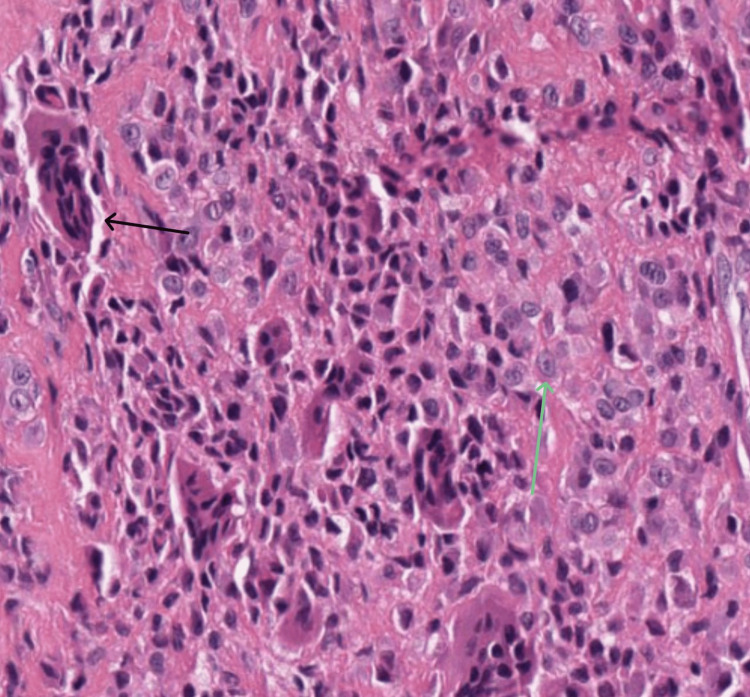
Hematoxylin and eosin staining at high resolution The black arrow indicates a giant cell and the green arrow indicates large epithelioid cells with amphophilic cytoplasm and rounded vesicular nuclei

After the excision of the swelling, the patient was intraoperatively assessed for motor function. The patient was noted to have a normal motion range of the toes and the right foot. The histopathology report showed histopathological features suggestive of benign fibrous histiocytoma, also called a 'giant cell tumor of tendon sheath', as shown in Figure 6.

The patient was managed postoperatively with antibiotics, analgesics, and lower limb physiotherapy. The patient was relieved of preoperative pain, tingling, and numbness without any motor and sensory loss. After discussing the case in tumor board discussion, no further treatment was needed for the patient. Complete suture removal was done on postoperative day 10, and the patient was discharged. The patient was followed up at one month, which showed a healthy scar line, with no motor or sensory loss, and no restricted movements. Nutritional build-up was also noted to be good.

## Discussion

GDTTS offers comprehensive insights into its clinical and pathological characteristics. GCTTS are benign, with special cytological features distinguishing these from other clinical conditions. Its evolution was discussed by Ozalp et al. in 2004 [7,8]. This is a case of atypical presentation exemplifying GCTTS in a 54-year-old female patient presenting with a swelling in the right foot as a benign yet locally aggressive lesion. This case is notable for the tumor’s unusual location in the foot, as GCTTS more commonly affects the hands. The presence of GCTTS on the foot is not a common observation [1,7,8]. Ushijima et al. distinguished between GCTTS in digits and large joints, highlighting the presence of specific cell types in these tumors. Different cell types involved are lipid-laden cells, mononuclear cells, foamy macrophages, multinucleated giant cells, hemosiderophages, and osteoclast-like giant cells. It has been characterized by its well-defined growth along tendons. These tumors can have a differential diagnosis based on MRI findings, which can be helpful to address their aggressive nature [5,9].

This case reinforces the role of comprehensive diagnostic modalities, including X-ray, ultrasonography, and possibly MRI, in preoperative planning, ensuring a clear understanding of the tumor’s extent and relation to surrounding structures. The successful identification and complete surgical excision of the tumor and its capsule underline the critical importance of thorough clinical evaluation and precise surgical intervention in managing such cases. Intraoperatively, the characteristics of the tumor's origin between the flexor tendons and extensions adhering to the bone without causing erosion or invasion were consistent with the benign nature of GCTTS [7,9]. These findings also highlight the aggressive potential of GCTTS and warrant careful surgical dissection to remove the specimen in toto; otherwise, incomplete excision can lead to recurrence, a known complication of GCTTS. A multidisciplinary treatment approach has been emphasized in managing the locally aggressive nature of GCTTS in the foot and ankle [1]. GCTTS is treated surgically with local synovectomy, location-specific therapies, with supportive therapy such as radiation therapy and systemic therapy. A complete surgical excision has been marked as the gold standard treatment for GCTTS, noting recurrence as a significant concern influenced by factors like incomplete excision and joint involvement [6]. Incomplete excision has been identified as a substantial cause of recurrence in postsurgical excision of GCTTS [10]. Research evidence highlights high recurrence rates of GCTTS in the upper limb, and adjuvant therapy has been advised in cases of high risk, with research demonstrating a decline in incidence rates in cases subjected to postoperative radiotherapy [4,11]. However, there are recommendations for a conservative approach to GCTTS attributed to its well-encapsulated nature, which highly facilitates tumor enucleation. These characteristics should be considered during diagnosis and treatment [12]. Follow-up of GCTTS is crucial to monitor for potential recurrences and ensure optimal treatment outcomes. Regular monitoring by radiological imaging tools can be recommended after three to six months of the procedure to keep a check on the recurrence. The frequency and duration of follow-up depend on several factors, including GCTTS type, its location, and other co-morbidities.

## Conclusions

Overall, this case contributes valuable insights into the management of GCTTS, particularly in atypical locations like the foot. It underscores the necessity of a meticulous approach in surgical excision, balanced with the preservation of surrounding anatomical structures, to achieve optimal outcomes and preserve the functionality of the foot. Postoperative prognosis is expected to be favorable, with a reduced recurrence risk and good function recovery, provided that close postoperative monitoring and follow-up are conducted to promptly address any signs of recurrence.
